# A Peptoid-Based Fluorescent Sensor for Cyanide Detection

**DOI:** 10.3390/molecules21030339

**Published:** 2016-03-10

**Authors:** Bumhee Lim, Jeeyeon Lee

**Affiliations:** College of Pharmacy, Research Institute of Pharmaceutical Sciences, Seoul National University, 1 Gwanak-ro, Gwanak-gu, Seoul 151-742, Korea; hihiboom@snu.ac.kr

**Keywords:** fluorescence, peptoid, cyanide sensor

## Abstract

Peptoids, *N*-substituted glycine oligomers, are versatile peptidomimetics with diverse biomedical applications. However, strategies to the development of novel fluorescent peptoids as chemical sensors have not been extensively explored, yet. Here, we synthesized a novel peptoid-based fluorescent probe in which a coumarin moiety was incorporated via copper(I)-catalyzed azide-alkyne cycloaddition reaction. Fluorescence of the newly generated coumarin-peptoid was dramatically quenched upon coordination of the Cu^2+^ ion, and the resulting peptoid-Cu^2+^ complex exhibited significant Turn-ON fluorescence following the addition of CN^−^. The rapid and reversible response, combined with cyanide selectivity of the synthesized peptoid, reflects a multistep photo-process and supports its utility as a new type of CN^−^ sensor.

## 1. Introduction

Peptoids, *N*-substituted glycine oligomers, are versatile mimics of peptides and are known to interact with various biological targets [1,2]. The proteolytic stability of peptoids, along with improved cell permeability relative to peptides, has led to their wide application as ligands for biological targets in various biomedical fields [3,4,5,6,7,8]. However, their significant conformational flexibility, combined with lack of hydrogen bonding ability, impose a major challenge for exploitation of peptoids in the drug discovery program.

Extensive studies have been devoted to enhancing the structural and functional capabilities of peptoids (including foldamers) [9,10,11]. Construction of peptoids with homogeneously-populated conformers remains a difficult task due to the low energy barrier of *cis*/*trans-*isomerization [12,13,14]. In particular, the development of new strategies to constrain flexible peptoids that adopt certain secondary structures is still challenging. To expand the applications of peptoids, understanding the physicochemical propensity that controls optimal conformations is essential. One of the methods to accomplish this goal is to attach fluorophores to the peptoid backbone and monitor interactions [15].

The 1,2,3-triazolyl group synthesized by the copper(I) catalyzed azide-alkyne [3+2] cycloaddition reaction coordinates with metal ions, with N_2_ or N_3_ directly involved in coordination [16,17]. Owing to their metal binding capacity [9,18,19,20,21,22,23,24,25,26,27], combined with ease of incorporation of fluorophore modules using solid-phase synthesis, peptoids present a good platform for the development of novel fluorescence sensors or functional peptidomimetics. In addition, understanding metal binding interactions with proteins is critical because human bodies are under the tight regulation of metal concentration inside and outside cells [28]. As a proof of concept to demonstrate a fluorescent peptoid sensor that can mimic the copper binding motif in many proteins, a simplified form of scaffold with functional diversities needs be explored.

Coumarins are one of the extensively used fluorophores because of their small size, high quantum yield, and efficient membrane permeability. Metal-complexed coumarin derivatives are versatile sensor molecules [29,30,31,32,33,34]. Recently, increasing numbers of triazolyl coumarin motifs have been utilized for various biological applications, such as the development of anti-inflammatory [35], anticancer [36], and antibacterial agents [37]. These compounds have also been explored as selective chemosensors [35,38,39,40,41]. In addition to the various advantages, including fast response and high sensitivity, development of new fluorescence sensor molecules for selective detection of ions is an active focus of biochemical research. Among the anions, efficient detection of the cyanide anion is particular interest due to severe toxicity issues related to human health [42,43,44,45]. In addition to wide industrial applications, the release of CN^−^ is harmful to the environment. So far, limited studies have explored the development and utility of peptoid-derived fluorescence sensors [15,46,47].

In this study, we have reported the synthesis and characterization of a novel peptoid-based fluorescence probe. The coumarin moiety was incorporated into the peptoid backbone via copper(I) catalyzed azide-alkyne [3+2] cycloaddition reaction. The fluorescence of the coumarin-peptoid was quenched by coordination with Cu^2+^ ion. The peptoid-Cu^2+^ complex exhibited significant Turn-ON fluorescence upon the addition of CN^−^. In view of its utility in rapid, selective, and reversible detection of CN^−^, our newly-generated peptoid molecule may serve as a novel type of CN^−^ sensor.

## 2. Results

### 2.1. Synthesis of Coumarin-Attached Peptoids

**Figure 1:**
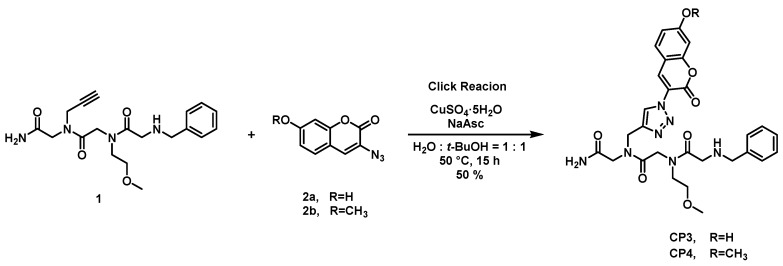
Synthesis of coumarin-attached peptoids.

The synthesis of coumarin-attached peptoids is depicted in Figure 1. We expected that the coumarin unit at residue 1 would have a π-π interaction with any aromatic unit attached at residue 3. We limited the size of the peptoid as a trimer in our design, which would be a minimal length in terms of the number of atoms for metal complexation while avoiding the complicated NMR characterization of peptoids due to the presence of many rotamers.

The amine building block containing the acetylene group was incorporated using the standard solid phase method to accommodate alkyne peptoid **1** [48]. The peptoid was subsequently connected with azido-coumarin **2** via Cu(I)-catalyzed Huisgen 1,3-dipolar cycloaddition (CuAAC) using copper sulfate and sodium ascorbate in *tert*-butyl alcohol/water solution (*v*/*v* = 1:1) [49].

### 2.2. Spectroscopic Features of Free ***CP3***

**Figure 2:**
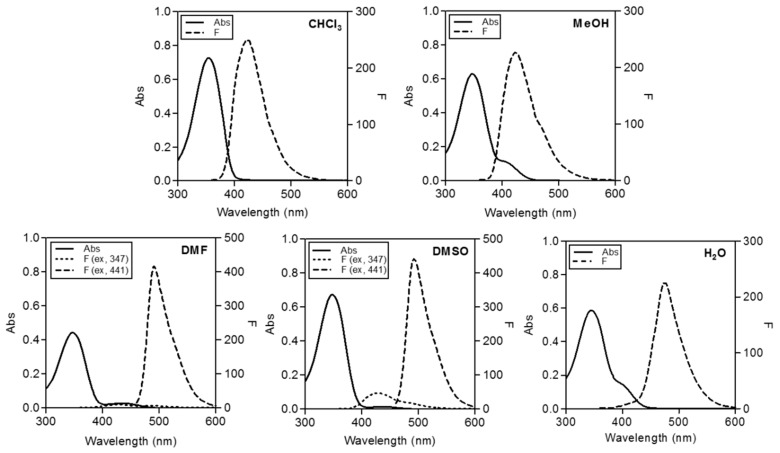
Spectroscopic features of **CP3** (1 μM for fluorescence, 50 μM for absorbance) in various solvents.

Figure 2 shows the absorption and emission spectra of the free form of **CP3**. UV spectra disclosed maximum absorption at around 350 nm in most solvents, with additional absorption bands at longer wavelengths, as shown in Table 1. In aprotic polar solvents, such as DMF and DMSO, typical coumarin emission observed at ~430 nm decreased and a new emission band at longer wavelengths was observed*.* **CP3** displayed dual emission. Excitation at 347 nm produced emission spectra with a maximum wavelength at 429 nm whereas excitation at 441 nm led to an additional emission band maximized at ~492 nm. The emission band at 429 nm represents 7-OH* of 7-hydroxycoumarin, while that at ~492 nm is likely to be 7-O^−^* of coumarin in the excited state. This dual emission was previously reported to occur due to the equilibrium between 7-OH* and 7-O^−^* of coumarin derivatives in the excited states [50].

### 2.3. Fluorescence Changes with Formation of the ***CP3***-Cu^2+^ Complex

**Figure 3:**
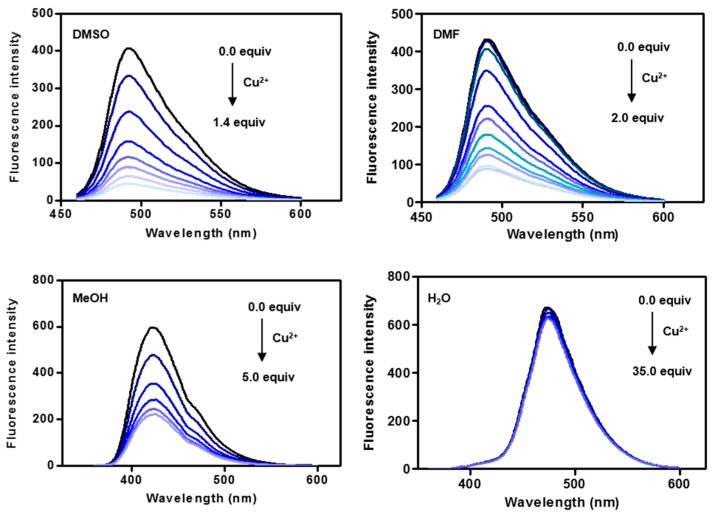
Changes in emission spectra upon addition of Cu^2+^. **CP3** (1 μM) was titrated against increasing amounts of Cu^2+^ (0 to 35 µM) in various solvents with excitation at 441 nm for DMSO and DMF, 347 nm for MeOH and 344 nm for H_2_O, respectively.

First, we explored the metal coordination ability of the coumarin-peptoid **CP3** in various solvents using fluorescence spectroscopy. The changes in emission spectra induced upon the addition of Cu^2+^ are shown in Figure 3. The maximal emission band at 492 nm was markedly decreased in the presence of increasing concentrations of Cu^2+^ in DMF. A similar trend was observed upon addition of Cu^2+^ to **CP3** solution in DMSO. This decrease in fluorescence appears to be related to photoinduced electron transfer (PET) based on dramatic fluorescent intensity changes with no spectral shift [51,52].

In the presence of protic solvents, such as H_2_O and methanol, the quenching effect upon addition of Cu^2+^ was different. No spectral changes were observed in water, while the addition of Cu^2+^ resulted in a decrease of emission at 423 nm in methanol, but to a lesser extent than in DMF/DMSO. Even excess amounts of Cu^2+^ ion (35 equiv.) did not induce fluorescence quenching in water, indicating a constrained peptoid structure, presumably due to π-π stacking between coumarin and the benzene unit (Figure S1).

**Figure 4:**
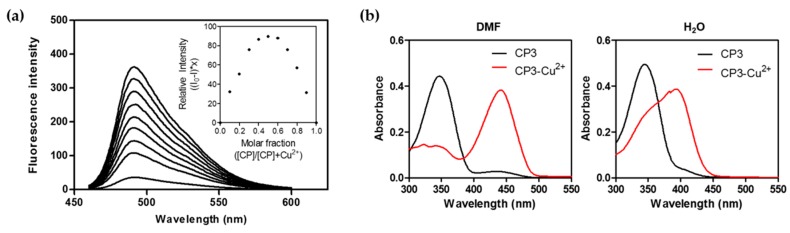
Spectroscopic features of **CP3** in the presence of Cu^2+^. (**a**) Job plot data on emission changes upon addition of Cu^2+^ in DMF at 492 nm (λ_ex_ = 441 nm). The X-axis represents the molar fraction of **CP3** and the Y-axis represents relative fluorescence intensity at an invariant total concentration of 1 μM; (**b**) UV-Vis spectra of **CP3** (50 μM) and **CP3**-Cu^2+^ (50 μM) in DMF and H_2_O.

Considering the chelation-induced fluorescence changes in **CP3** upon addition of Cu^2+^, we determined the stoichiometry of the peptoid-Cu^2+^ complex. Figure 4 shows Job plot analysis demonstrating a maximum at a mole fraction of 0.5, suggesting a 1:1 stoichiometry for the **CP3**-Cu^2+^ complex. The binding constant (*K*_d_) of the peptoid-Cu^2+^ complex was 0.64 ± 0.093 μM in DMF and 0.65 ± 0.36 μM in MeOH:CHCl_3_ (*v*/*v* = 1:5), as measured from an equilibrium binding titration experiment (Figure S2, Table S1). Changes in absorption spectra between free and Cu^2+^-complexed **CP3** are shown in Figure 4b.

### 2.4. The ***CP3***-Cu^2+^ Complex as a Cyanide Sensor

**Figure 5:**
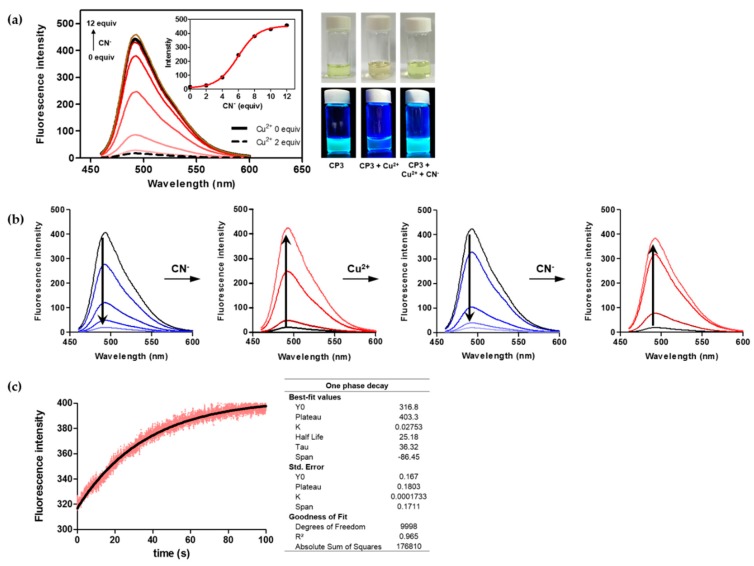
Fluorescence response of **CP3**-Cu^2+^ to CN^−^. (**a**) Emission increase upon addition of CN^−^ (0 to 12 μM) to **CP3**-Cu^2+^ (1 μM) in DMSO (λ_ex_ = 441 nm). Insert : changes in fluorescence intensity of the solution measured at 492 nm; (**b**) rapid and reversible ON/OFF switching property of **CP3**-Cu^2+^ (1 μM) with CN^−^ (0 to 12 μM) in DMSO; and (**c**) measurement of the time course for the cyanide detection by **CP3**-Cu^2+^ (1 μM) as monitored by fluorescence increase in DMSO. The black line represents the fit of the data.

Next, we examined whether the peptoid-Cu^2+^ complex is dissociated in the presence of anions. As shown in Figure 5a the peptoid-Cu^2+^ complex exhibited Turn-ON fluorescence upon the addition of cyanide ions. Emission at 492 nm (solid black line) quenched in the presence of Cu^2+^ (dotted black line) was recovered by the addition of increasing amounts of cyanide. Turn-ON fluorescence is attributable to the formation of a [Cu(CN)_x_]^n−^ complex [53], resulting in release of the free form of fluorescent coumarin-peptoid. Interestingly, this fluorescence change was reversed when Cu^2+^ ion was re-added to the same solution (Figure 5b). Notably, the ON/OFF switch response of **CP3** was reproducible and rapid. All fluorescence emission spectra were recorded after each addition of the ion solutions was equilibrated for 1 min. Figure 5c demonstrates the kinetic profile of the fluorescence intensity increase at 492 nm when 5 μM of the cyanide anion was added to the 1 μM of **CP3**-Cu^2+^ complex. The kinetic trace was best fit to a single exponentional rate equation. The rate constant was measured as the increase in fluorescence intensity was 0.028 s^−1^, which corresponds to a half-life *(t_1_*_/2_) of 25.2 s. The data demonstrate a rapid sensing of **CP3**-Cu^2+^ complex for cyanide ion.

**Figure 6:**
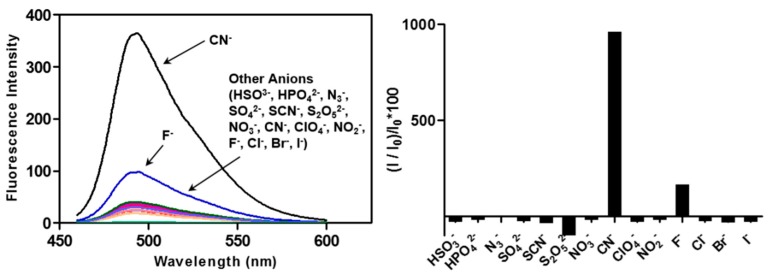
Selectivity of **CP3**-Cu^2+^ for cyanide among various anions examined in DMSO solution. Changes in emission spectra of **CP3**-Cu^2+^ (1 μM) in DMSO upon addition of 20 equiv. of various anions (CN^−^, HSO_3_^−^, HPO_4_^2−^, N_3_^−^, SO_4_^2−^, SCN^−^, S_2_O_5_^2−^, NO_3_^−^, ClO_4_^−^, NO_2_^−^, F^−^, Cl^−^, Br^−^, I^−^) (λ_ex_ = 441 nm).

To determine the selectivity of peptoid-Cu^2+^ in anion sensing, we measured the recovered fluorescence increase following binding to various anions, including halogens, phosphate, azide, and sulfur anions (Figure 6). Among these, only the cyanide anion induced a fluorescence increase for **CP3**-Cu^2+^. The observed selectivity for the cyanide anion along with the rapid and reversible sensing properties of peptoid-Cu^2+^ supports its potential utility as a new class of cyanide sensor.

The 1-D NMR spectra of free **CP3** and **CP3**-Cu^2+^ complex were compared, as shown in Figure S3a. In the complex, peaks in the aromatic proton region were shifted upfield. The data support the formation of 2-hydroxy coumarin via tautomerization, which may be promoted by Cu^2+^ coordination at the 2-hydroxy position.

### 2.5. ***CP4*** Displays Different Spectroscopic Features to ***CP3***

**Figure 7:**
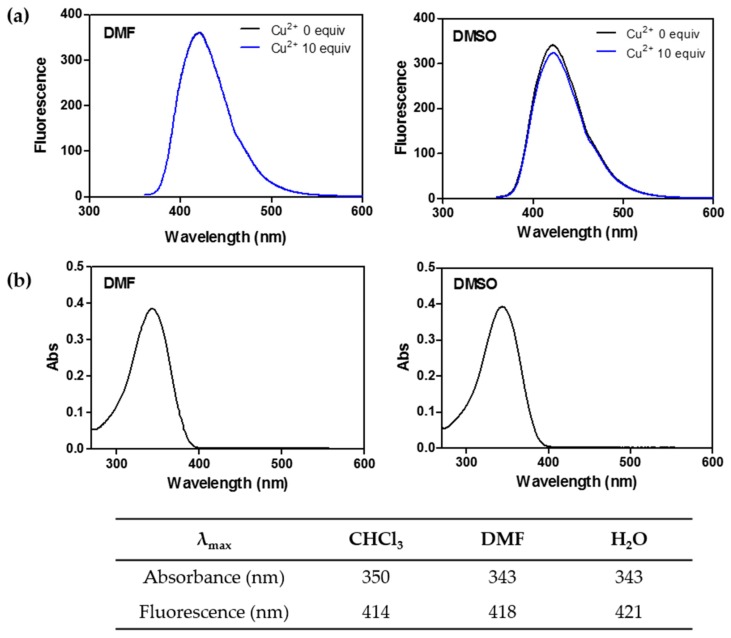
Spectroscopic features of 7-methoxy coumarin-peptoid (**CP4**) as a control. (**a**) fluorescence spectra of **CP4** (1 μM) in response to the presence of Cu^2+^ (0 to 10 μM) in DMF and DMSO (λ_ex_ = 343 nm, λ_em_ = 418 nm, slit widths = 3 nm for excitation and 5 nm for emission); (**b**) absorption spectra of **CP4** (50 μM) in DMF and DMSO (λ_max_ = 343 nm).

We additionally examined the spectroscopic features of the 7-methoxy analogue, **CP4**, to gain further insights into the mechanism of coumarin-peptoid. As shown in Figure 7, the emission peak at 492 nm observed with **CP3** in DMF/DMSO was absent, resulting in a sole emission band at 418 nm. Furthermore, no fluorescence changes were observed, even upon addition of 10 equiv. of Cu^2+^ ion either in DMF or DMSO solvent (Figure 7). This result supports our hypothesis that the hydroxy group is critical for metal coordination with Cu^2+^, which is facilitated by the electron density redistribution within **CP3**.

## 3. Discussion

The unique photophysical properties of hydroxycoumarin have been documented, both spectroscopically and theoretically [54,55,56,57]. Here, we constructed a peptoid coordinated with Cu^2+^ where a fluorescent coumarin module was attached via click chemistry.

**Figure 8:**
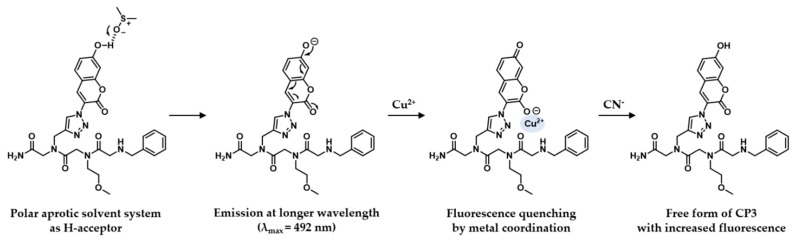
Proposed mechanism underlying fluorescence sensing of coumarin peptoids.

Figure 8 depicts the proposed mechanism of fluorescence sensing based on our observations with **CP3**. Excited state proton transfer (ESPT) appears to be related to the shift in electron density from hydroxyl to carbonyl group in 7-hydroxycoumarin. Redistribution of the electron density within **CP3** upon photo-excitation explains the enhanced acidity of 7-hydroxycoumarin moiety, triggering photoinduced proton transfer to the solvent [54]. The p*K*_a_ of the 7-hydroxy group of coumarin is ~7.7 in the ground state, while photoexcitation enhances the acidity of the hydrogen-bonded proton to ~0.45 in the S_1_ state [56]. The resulting anion of **CP3** is highly emissive with a maximum peak at 492 nm.

Photoinduced electron transfer (PET) additionally occurs in the (tautomerized, 2O^−^) anionic coumarin fluorophore in the presence of Cu^2+^, thereby inducing fluorescence quenching at 492 nm. PET within the peptoid-Cu^2+^ complex was featured by large fluorescence intensity changes with no spectral shift. This OFF response with Cu^2+^ was subsequently reversed by the addition of cyanide ion, leading to an OFF-ON fluorescent response. This phenomenon is attributable to the formation of a stable [Cu(CN)_x_]^n−^ complex [58,59]. The observed photo-driven processes were rapid, switchable, and reproducible.

Hydroxycoumarin-based chemosensors have been reported previously [40,55], but our molecule utilizes the unique photophysical characteristic of photo-excited enol-to-keto conversion in 7-hydroxycoumarin. In the previous report, the anion of 7-hydroxycoumarin was shown to be directly involved in metal coordination [55], whereas photo-excited enol-to-keto conversion resulted in metal coordination with the hydroxy anion at the C2 position in our molecule. Coumarin was used as a fluorescence reporter as well as coordination ligand. The excited state of 7-hydroxy-4-methylcoumarin is reported to favor the keto form, with the S_1_ potential energy minima of the enol/keto form separated by a 17–20 kcal/mol energy barrier. In the ground state, the enol form is more stable whereas the keto form is more stable by 0.6 kcal/mol in the S_1_ state [56]. Our NMR results were consistent with the reported enol-to-ketone transformation in the presence of Cu^2+^ ion, revealing upfield-shifted peaks in the aromatic region (Figure S3a).

Cu^2+^ is known to have strong paramagnetic properties due to the faster electron-relaxation time (Ts) of Cu^2+^. Therefore, the Cu^2+^ complex is hard to characterize by NMR. Various studies have reported severe line broadening of the Cu^2+^ binding to metalloproteins and used other experimental methods than NMR (including CD, EPR, UV-Vis, X-ray crystallography, *etc.*) [28,60,61]. Initially, we tried to get a crystal for X-ray crystallography, which will unequivocally reveal the metal-complexed peptoid, but the crystal was not obtained. The absorption spectra are, in general, critical to verify the differences in the binding of ligands to metal. However, the absorption spectra of Cu^2+^ solution were overlapped with those of **CP3** in DMSO and DMF (λ_max_ = 300 nm in DMSO; λ_max_ = ~280 in DMF), which imposed difficulties in obtaining reliable titration data based on the absorbance. Even though we observed absorption spectral changes between free **CP3** and the **CP3**-Cu^2+^ complex (Figure 4b), the only binding stoichiometry that we determined for the **CP3**-Cu^2+^ complex was from the fluorescence data. This issue may be resolved once the structural information of the **CP3**-metal complex is obtained.

**CP3** in the aqueous system does not exhibit fluorescence quenching by Cu^2+^, possibly due to the constrained conformation resulting from π-π stacking interactions, which does not afford optimal geometry for coordination with Cu^2+^. We performed a simulated annealing analysis followed by energy minimization of each conformer. An ensemble of energy-minimized structures with the lowest E values is depicted in Figure S1. The trans-trans rotamer showed the lowest energy (9.7 kcal/mol), furnishing π-π stacking interactions between the benzene ring and coumarin unit.

In summary, we have developed peptoid-Cu^2+^ complex that effectively acts as a CN^−^ sensor. Photo induced proton transfer resulted in conversion of the 7-hydroxycoumarin moiety to its tautomeric form, facilitating metal coordination within the peptoid. The current form of the peptoid-Cu^2+^ complex only working in aprotic polar solvents represents a drawback for practical applications, which requires more systematic approaches to develop peptoid sensors working in aqueous solution. Although detailed mechanistic analyses are yet to be conducted, our understanding on the peptoid-based sensor design can be enriched by the knowledge obtained in the present work with a newly developed coumarin-attached peptoid-Cu^2+^ probe*.*

## 4. Experimental Section

### 4.1. General

Unless specified, all reagents and starting materials were ACS grade or higher and were used without further purification. All reactions were monitored by thin-layer chromatography on a TLC silica gel 60 F254 plate (Merck, Darmstadt, Germany), and compounds were visualized under UV light (254, 365 nm, VL-4.LC, Vilber Lourmat, Eberhardzell, Germany). Flash column chromatography was performed using ZEOprep silica gel (230~400 mesh, Zeochem, Lake Zurich, Switzerland) with hexane, ethyl acetate, dichloromethane, and methanol as eluents. ^1^H (300, 800 MHz) and ^13^C-NMR (75, 200 MHz) spectra were recorded on a GEMINI 2000 (VARIAN, Palo Alto, CA, USA) and FT-NMR Avance III HD (Bruker, Billerica, MA, USA). Chemical shifts (δ) are reported in parts per million (ppm) and coupling constants (*J*) are given in hertz (Hz). All ESI-MS were undertaken on a 6130 Single Quadrupole LC/MS (Agilent Technologies, Santa Clara, CA, USA) and high-resolution mass spectra (HR-MS) were acquired under fast atom bombardments (FAB) condition on a JMS-700 MStation (JEOL, Tokyo, Japan). UV-Vis and fluorescence spectra were obtained using a Lambda 25 (Perkin Elmer, Waltham, MA, USA) and FP-6500 (Jasco, Tokyo, Japan). High-performance liquid chromatography (HPLC) analysis was performed on a YL9100 reversed-phase HPLC (Younglin, Anyang, South Korea).

### 4.2. Synthesis of N-(2-Amino-2-oxoethyl)-2-(2-(benzylamino)-N-(2-methoxyethyl)acetamido)-N-(prop-2-yn-1-yl)acetamide (Solid Phase Peptide Synthesis) *(**1**)*

Peptoid oligomer was synthesized on MBHA resin (0.43 mmol/g) using a conventional peptoid synthesis protocol to generate an amide group at the *C*-terminus of the peptoids [2]. The Fmoc protected resin was swollen in *N*,*N*-dimethylformamide (DMF) for 30 min before starting oligomer synthesis. The Fmoc group was removed with 20% piperidine in DMF. Acylations using 1 M bromoacetic acid (BrAA, 10 equiv.) and 1 M *N*,*N*′-diisopropylcarbodiimide (DIC, 10 equiv.) in DMF followed by nucleophilic displacement step using 0.5–2 M amine (propargyl amine, 2-methoxyethylamine, benzylamine) in DMF were repeated until desired peptoid was obtained. The resin was cleaved with 95% trifluoroacetic acid (TFA) in H_2_O for 1 h with occasional agitation (180 rpm). The filtered solution was purged with N_2_ to remove TFA. The crude compounds were dissolved in 50% acetonitrile/H_2_O and lyophilized two times to remove residual trifluoroacetic acid (HPLC purity: 93.6%). ^1^H-NMR (300 MHz, DMSO-*d*_6_): δ 9.32 (bs, s, 2H), 7.51–7.40 (m, 5H), 4.46/4.42* (2 × s, 1H), 4.27–4.22 (m, 2H), 4.13–4.07 (m, 6H), 3.93/3.89* (2 × s, 1H), 3.8 (bs, s, 1H), 3.46–3.40 (m, 4H) 3.30/3.25* (2 × t, 1H), 3.19 (m, 3H) (rotamer peaks*). ^13^C-NMR (75 MHz, DMSO-*d*_6_): δ 169.7, 169.5*, 169.3*, 169.1*, 168.4, 168.0*, 167.8*, 167.4*, 166.5, 165.9*, 158.8, 158.4*, 158.0*, 157.5*, 131.6, 131.5*, 130.3, 130.23*, 130.17, 129.1, 128.7, 79.1, 78.9*, 78.8*, 78.5*, 76.1, 75.7*, 75.2*, 74.9*, 69.5, 69.4*, 69.3*, 69.2*, 58.34, 58.30*, 58.1, 58.0*, 50.1, 49.9*, 49.0*, 48.9*, 48.6*, 48.1, 48.0*, 47.4, 46.9*, 46.7*, 46.5, 46.2*, 37.3, 37.2*, 35.7*, 35.4* (rotamer peaks*). HR-MS (*m/z*): 375.2034 (calculated for C_19_H_27_N_4_O_4_ [M + H]^+^, 375.2032).

### 4.3. Synthesis of 3-Azido-7-hydroxycoumarin *(**2a**)* and 3-Azido-7-methoxycoumarin *(**2b**)*

The compounds **2a** and **2b** were synthesized according to the reported method [49].

*3-Azido-7-hydroxycoumarin* (**2a**)*.* ^1^H-NMR (300 MHz, MeOH-*d*_4_): δ 7.40 (s, 1H). 7.37 (d, *J* = 2.1 Hz, 1H), 6.80 (d, *J* = 2.4 Hz, 1H), 6.77 (d, *J* = 2.4 Hz, 1H), 6.72 (d, *J* = 2.1 Hz, 1H).

*3-Azido-7-methoxycoumarin* (**2b**)*.* ^1^H-NMR (300 MHz, CDCl_3_): δ 7.31 (d, *J* = 8.4 Hz, 1H), 7.18 (s, 1H), 6.89–6.84 (m, 2H), 3.87 (s, 3H).

### 4.4. Synthesis of N-(2-Amino-2-oxoethyl)-2-(2-(benzylamino)-N-(2-methoxyethyl)acetamido)-N-((1-(7-hydroxy-2-oxo-2h-chromen-3-yl)-1h-1,2,3-triazol-4-yl)methyl)acetamide (***CP3***)

Copper (II) sulfate pentahydrate (0.3 M in water, 35 μL, 0.0104 mmol) and sodium ascorbate (1 M in water, 42 μL, 0.0416 mmol) were added to a mixture of alkyne peptoid, **1** (39 mg, 0.104 mmol) and 3-Azido-7-hydroxycoumarin, **2a** (20 mg, 0.104 mmol) in water and *tert*-butyl alcohol (*v*/*v* = 1:1, 4 mL). The reaction mixture was stirred at room temperature for 24 h in the dark. After removing the solvent under reduced pressure, the crude material was purified by column chromatography (MeOH/DCM, 1:10) afforded **CP3** as a yellow solid (30 mg, 50%). ^1^H-NMR (800 MHz, DMSO-*d*_6_): δ 11.01 (bs, s, 1H), 9.24 (bs. s, 2H), 8.71/8.67*/8.47*/8.41* (4 × s, 1H), 8.61/8.59*/8.58* (4 × s, 1H), 7.77–7.75 (m, 1H), 7.50–7.39 (m, 5H), 6.92 (d, *J* = 8.4 Hz, 1H), 6.87 (d, *J* = 8.7 Hz, 1H), 4.72/4.71* (2 × s, 1H), 4.68/4.62* (2 × s, 1H), 4.61/4.59* (2 × s, 1H), 4.30/4.26* (2 × s, 1H), 4.13 (bs, s, 2H), 4.08 (m, 2H), 3.88 (d, *J* = 7.4 Hz, 1H), 3.85 (bs, m, 1H), 3.50–3.46 (m, 2H), 3.44–3.40 (m, 2H), 3.19 (dd, *J_1_* = 13.3, *J_2_* = 2.7 Hz, 3H) (rotamer peaks*). ^13^C-NMR (200 MHz, DMSO-*d*_6_): δ 169.88, 169.65*, 169.57*, 169.39*, 168.67, 168.11*, 168.12*, 167.57*, 166.47, 166.46*, 165.80*, 165.79*, 162.59, 162.57*, 162.54*, 162.51*, 158.05, 157.89*, 157.73*, 157.58*, 156.25, 156.23*, 156.21*, 154.66, 154.65*, 154.61*, 154.59*, 143.31, 143.14*, 143.10*, 142.85*, 136.33, 136.15*, 136.10*, 131.52, 131.43*, 131.39*, 130.98, 130.96*, 130.93*, 130.22, 130.18*, 130.10*, 129.01, 128.64, 128.61*, 128.59*, 124.64, 124.7*, 124.38*, 124.24*, 119.17, 119.16*, 119.13*, 114.34, 114.33*, 110.27, 110.26*, 110.25*, 102.17, 102.14*, 69.31, 69.28*, 69.27*, 69.21*, 58.29, 58.25*, 57.97, 57.95*, 50.16, 50.13*, 49.88*, 49.86*, 49.09, 48.99*, 48.84*, 48.59, 48.21*, 47.29, 47.26*, 46.95*, 46.58*, 46.52*, 46.50*, 46.25*, 46.22*, 42.34, 41.87*, 41.59* (rotamer peaks*). HR-MS (*m/z*): 578.2368 (calculated for C_28_H_32_N_7_O_7_ [M + H]^+^, 578.2363). ^1^H- and ^13^C-NMR spectra of **CP3** are available in the Supplementary Materials (Figure S4).

### 4.5. Synthesis of N-(2-Amino-2-oxoethyl)-2-(2-(benzylamino)-N-(2-methoxyethyl)acetamido)-N-((1-(7-methoxy-2-oxo-2h-chromen-3-yl)-1h-1,2,3-triazol-4-yl)methyl)acetamide (***CP4***)

The synthetic procedure described above was used for the preparation of **CP3**. 3-Azido-7-methoxycoumarin, **2b** (14.5 mg, 0.067 mmol) and alkyne peptoid, **1** (25 mg, 0.067 mg) were used. The reaction mixture was purified by column chromatography to obtain 23 mg (58%) of **CP4** as a pale yellow solid. ^1^H-NMR (800 MHz, DMSO-*d*_6_): δ 9.21 (bs, s, 2H), 8.74/8.70*/8.50*/8.44* (4 × s, 1H), 8.67/8.66*/8.65* (4 × s, 1H), 7.87–7.85 (m, 1H), 7.49–7.39 (m, 5H), 7.18–7.16 (m, 1H), 7.09 (d, *J* = 8.6 Hz, 1H), 4.73/4.72* (2 × s, 1H), 4.68/4.63* (2 × s, 1H), 4.62/4.60* (2 × s, 1H), 4.30/4.27* (2 × s, 1H), 4.13 (d, *J* = 4.4 Hz, 2H), 4.08 (dd, *J*_1_ = 11.9, *J*_2_ = 3.9 Hz, 2H), 3.91 (d, *J* = 2.7, 3H), 3.88 (d, *J* = 8.3 Hz, 1H), 3.85 (d, *J* = 10.2 Hz, 1H), 3.50–3.46 (m, 2H), 3.44–3.40 (m, 2H), 3.19 (dd, *J_1_* = 13.6, *J*_2_ = 2.9 Hz, 3H) (rotamer peaks*). ^13^C-NMR (200 MHz, DMSO-*d*_6_): δ 169.87, 169.64*, 169.55*, 169.38*, 168.86, 168.12*, 168.02*, 167.57*, 166.51, 166.84*, 163.47, 163.45*, 163.43*, 163.40*, 157.68, 157.53*, 156.10, 156.09*, 154.53, 154.52*, 154.48*, 154.46*, 143.37, 143.20*, 143.16*, 142.91*, 135.80, 135.79*, 135.62*, 135.55*, 131.59, 131.47*, 130.64, 130.62*, 130.60*, 130.19, 130.15*, 130.08*, 129.00, 128.64, 128.60*, 128.59*, 124.62, 124.46*, 124.36*, 124.22*, 120.08, 120.07*, 120.05*, 113.57, 113.54*, 111.45, 111.44*, 111.43*, 100.73, 100.72*, 100.69*, 69.31, 69.27*, 69.21*, 58.29, 58.25*, 57.97, 57.95*, 56.20, 50.18, 50.15*, 49.90*, 49.88*, 49.11, 49.07*, 49.01*, 48.83*, 48.59*, 48.20*, 47.25, 46.94*, 46.51*, 46.49*, 46.27*, 46.25*, 42.35, 42.33*, 41.88*, 41.59* (rotamer peaks*). HR-MS (*m/z*): 592.2510 (calculated for C_29_H_34_N_7_O_7_ [M + H]^+^, 592.2520). ^1^H- and ^13^C-NMR spectra of **CP4** are available in the Supplementary Materials (Figure S5).

### 4.6. Spectroscopic Measurements

Fluorescence emission spectra were obtained at 20 °C using a JASCO FP-6500 spectrofluorometer (Jasco). The equilibrium binding titration experiments of peptoid with Cu^2+^ or other anions were performed as follows. 0.5–2 µL of metal or anion stock solutions (10 mM in DMSO) were added into 1 µM peptoid solutions in quartz cuvette, and the mixture was equilibrated for 1 min to ensure full binding before measurements. The slit widths used for measurement were 3 nm for both excitation and emission with medium sensitivity, but was adjusted depending on the strength of the fluorescence signal. The kinetic measurement was performed on the same instruments. The fluorescence intensity was recorded at 492 nm with 0.01 s time intervals without incubation time. For spectroscopic measurement, all stock solutions of peptoids and the sodium or potassium salts of anions (NaCN, NaHSO_3_, Na_2_HPO_4_, NaN_3_, Na_2_SO_4_, NaSCN, Na_2_S_2_O_5_, NaNO_3_, NaClO_4_, NaNO_2_, KF, KCl, KBr, KI) were prepared in DMSO (1% water). Absorption spectra were obtained at room temperature using a Lamda 20 UV-Vis spectrometer (Perkin Elmer, MA, USA) with 1.0 cm quartz cells. All measurements were triplicated. The **CP3**-Cu^2+^ complex for the absorption spectra was obtained by adding CuSO_4_ to **CP3** solution with stirring for 12 h at ambient temperature followed by purification described above. The ESI-MS spectrum of **CP3**-Cu^2+^ is available in Figure S6.

## Figures and Tables

**Table 1:** Spectroscopic values of **CP3**.

Wavelength	CHCl_3_	MeOH	DMF	DMSO	H_2_O
λ_ex_ (nm)	357	347	347/441	347/441	344
λ_em_ (nm)	425	423	429/492	429/492	475
Stokes shift (nm)	68	76	82/51	82/51	131

## Supplementary Materials

Supplementary materials can be accessed at: http://www.mdpi.com/1420-3049/21/3/339/s1.
