# Supplementary material for: A Peptoid-Based Fluorescent Sensor for Cyanide Detection
Source: Molecules. 2016 Mar 10;21(3):339. doi: 10.3390/molecules21030339 (PMC6273317; doi:10.3390/molecules21030339)
Supplement: Supplementary file 1 [file molecules-21-00339-s001.pdf]

Supplementary Materials: A Peptoid-Based Fluorescent Sensor for Cyanide Detection

Bumhee Lim and Jeeyeon Lee

Computational Methods

Molecular dynamics was carried out using the SYBYL-X2.1.1(Tripos Inc, St Louis, MO, USA) with the method of simulated annealing to find the lowest energy structures of CP3. The compound was heated at a temperature of 700 K for 1000 fs to allow high degree of randomization and was cooled down to a temperature of 200 K for 1000 fs for 10 cycles. The Tripos standard molecular mechanic force field and the Gasteiger–Huckel charge were used for simulation. The distance-dependent dielectric constant was set to a value of 1 and nonbonded cutoffs was set to a value of 8.0 Å. The annealed structures were finally subjected to the Powell energy minimization algorithm with a gradient 0.005 kcal mol^{-1}·$Å^{-1}$.

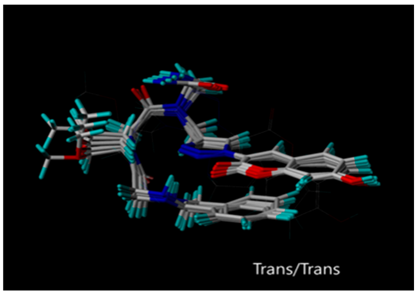

Figure S1. The ensemble of the lowest energy structures of coumarin-peptoid obtained from the simulated annealing method.

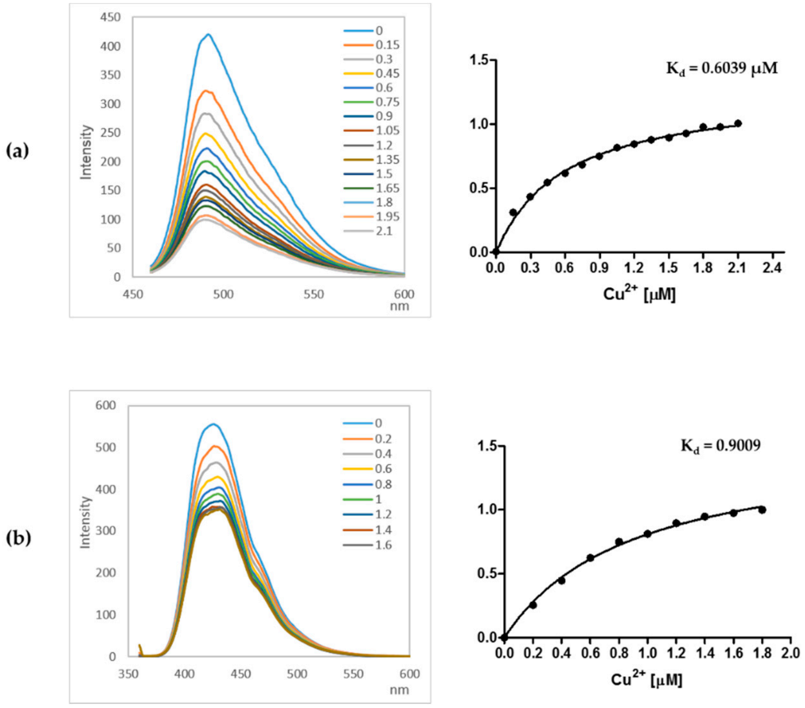

Figure S2. The binding constant (K_d) of the **CP3**-Cu^{2+} complex in DMF (**a**) and MeOH:$CHCl_3$ (1:5) (**b**) (λ_{ex} = 441 nm).

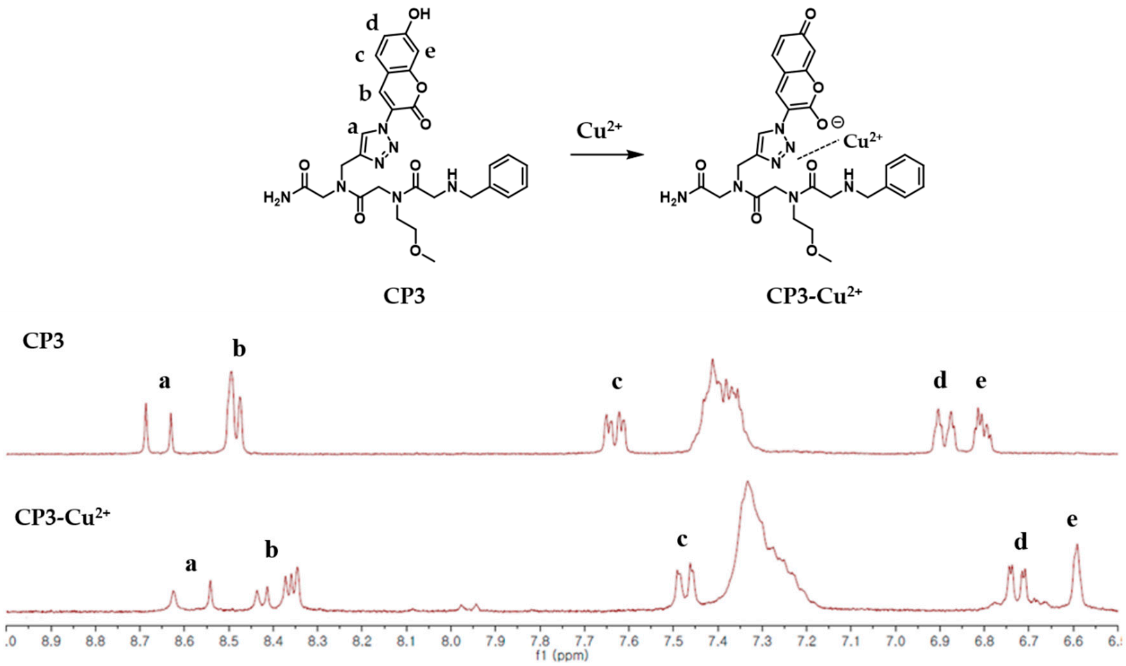

(**a**)

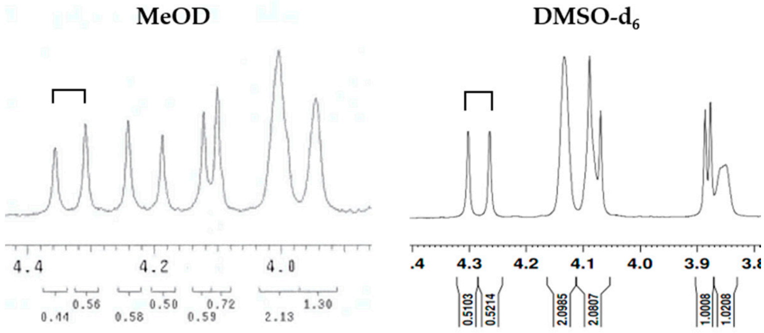

(**b**)

Figure S3. ^{1}H-NMR spectra of **CP3** and **CP3**-Cu^{2+} (**a**) ^{1}H-NMR spectral changes associated with formation of the **CP3**-Cu^{2+} complex. (300 MHz); (**b**) ^{1}H-NMR spectra of **CP3** in MeOD and DMSO-*d*6 (300 MHz). Marked peaks indicate rotamers.

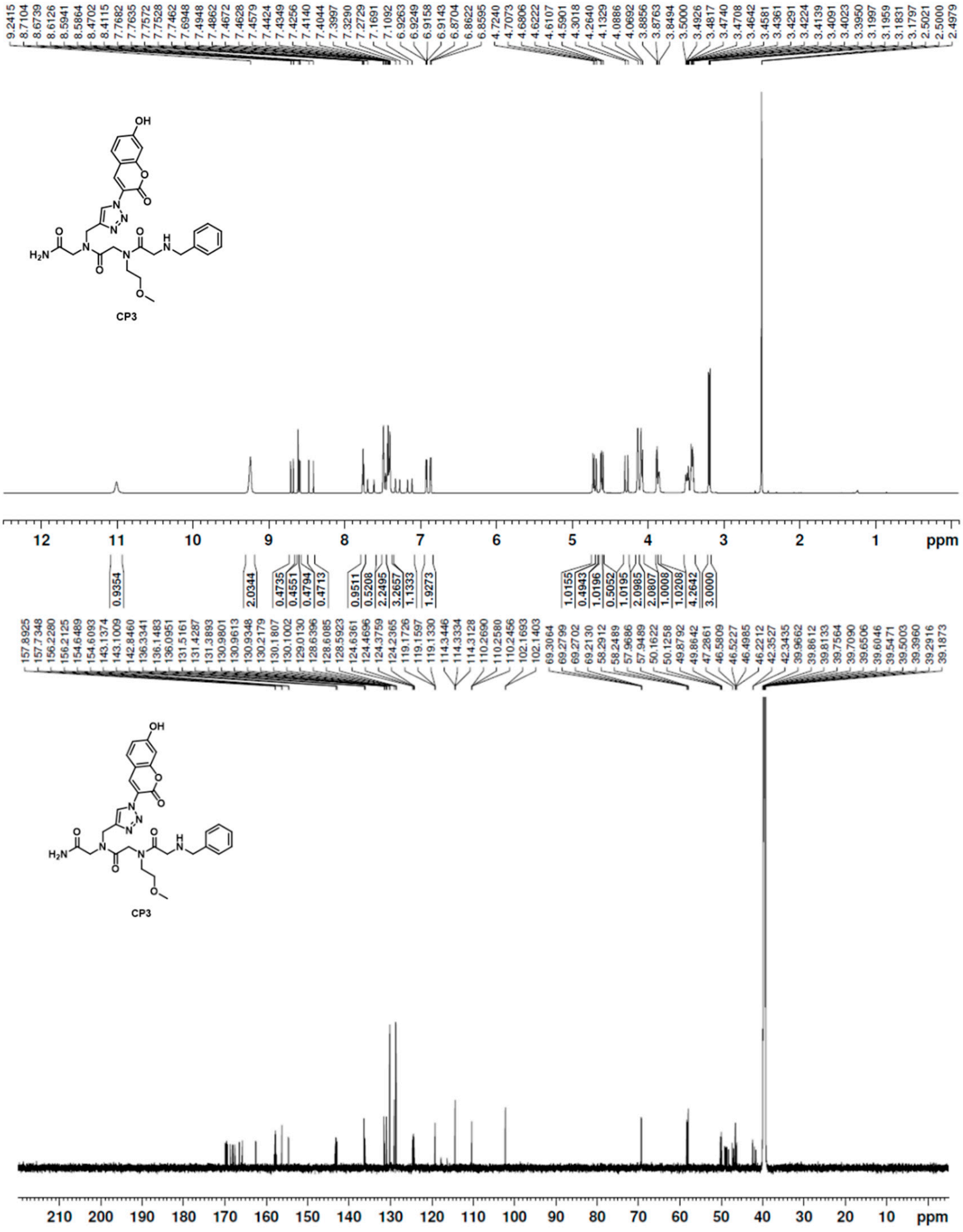

Figure S4. ^{1}H and ^{13}C-NMR spectra of compound **CP3** (DMSO-d_6).

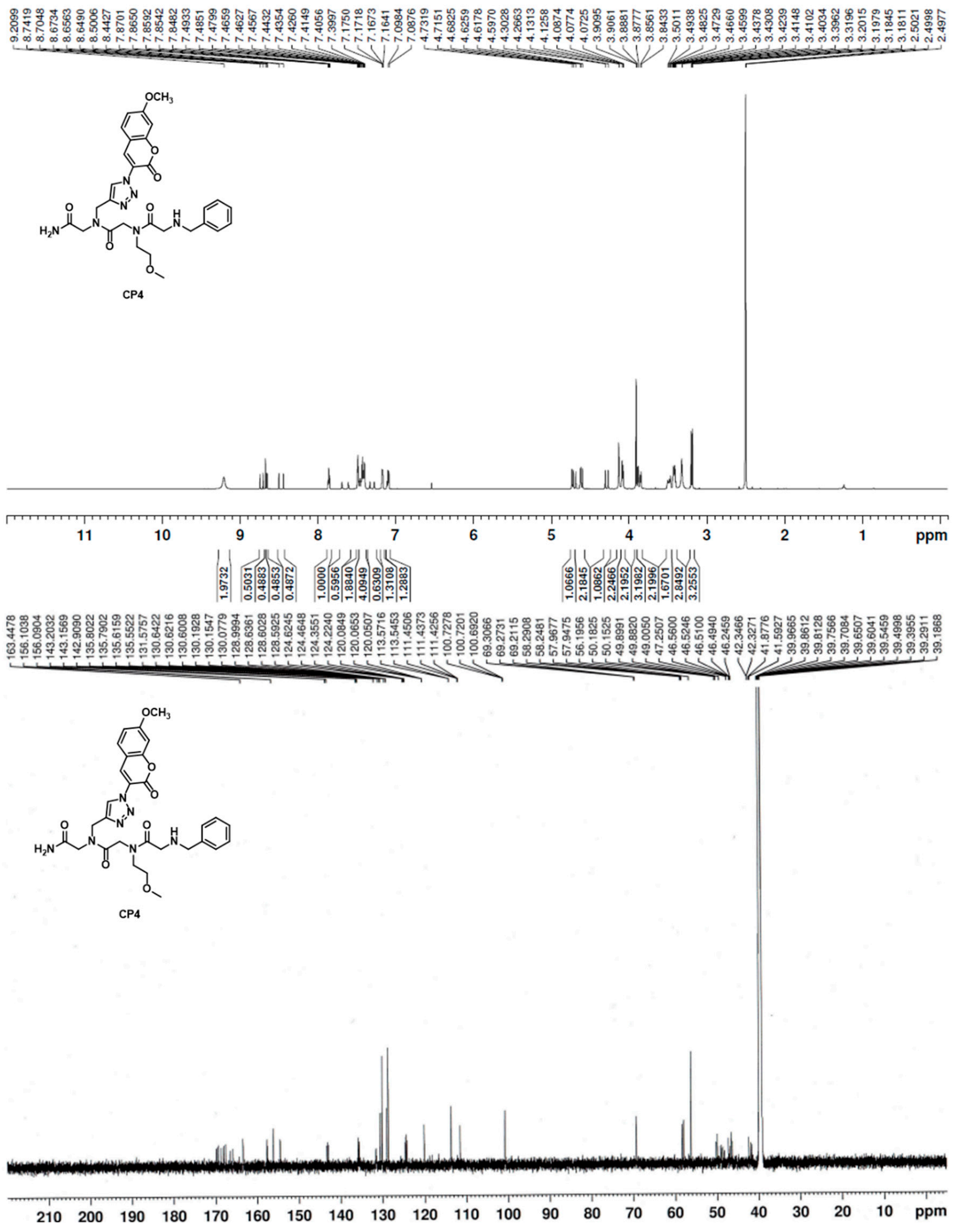

Figure S5. ^{1}H and ^{13}C-NMR spectra of compound **CP4** (DMSO-d_6).

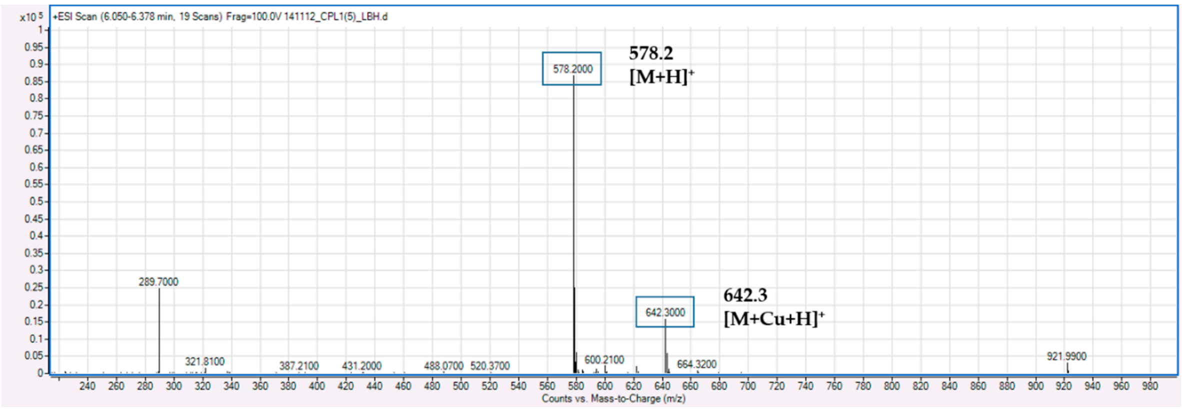

Figure S6. ESI-MS spectrum of **CP3**-Cu^{2+}.

Table S1. The binding constant (K_d) of the **CP3**-Cu^{2+}.

	Titration 1 (K_d)	Titration 2 (K_d)	Titration 3 (K_d)
DMF	0.5656	0.7426	0.6039
MeOH:$CHCl_3$	0.3940	0.9009	
